# Percentage of income spent on tobacco and intention to quit: a cross-sectional analysis of the JASTIS 2020 study

**DOI:** 10.1265/ehpm.22-00103

**Published:** 2022-12-03

**Authors:** Midori Takada, Takahiro Tabuchi, Hiroyasu Iso

**Affiliations:** 1 Cancer Control Center, Osaka International Cancer Institute, 3-1-69 Otemae, Chuo-ku, Osaka 541-8567, Japan; 2 Department of Public Health Medicine, Faculty of Medicine, and Health Services Research and Development Center, University of Tsukuba, 1-1-1 Tenno-dai, Tsukuba, Ibaraki 305-8575, Japan; 3 Public Health, Department of Social Medicine, Osaka University Graduate School of Medicine, 2-2 Yamadaoka, Suita, Osaka 565-0871, Japan

**Keywords:** Tobacco, Affordability, Cessation intentions, Heated tobacco products, Dual user, Japan

## Abstract

**Background:**

Existing indicators for the ease of purchasing tobacco did not reflect the actual amount smoked and individual income, and did not assess heated tobacco products (HTPs). This study assessed the percentage of income spent on tobacco, including combustible cigarettes and/or HTPs, at the individual level and its relation to quit intention.

**Methods:**

An internet-based self-reported questionnaire survey was conducted in 2020 as a part of the Japan Society and New Tobacco Internet Survey. A total of 954 smokers aged 15–72 years were analyzed. We calculated the percentage of income spent on tobacco according to income levels. A high percentage implies that tobacco is not easy to purchase. The odds ratios for quit intention according to three categories of percentage of income spent on tobacco (<1%, 1–5%, >5%) were calculated by multivariable logistic regression.

**Results:**

The percentage of income spent on tobacco was higher as income level was lower, especially for dual cigarette and HTP users; the percentages in the lowest/highest income group were 7.1%/1.2% for exclusive combustible cigarette smokers; 6.5%/1.1% for exclusive HTPs users; and 9.2%/1.3% for dual users. The adjusted odds ratios (95% confidence intervals) of quit intention among the >5% of income spent on tobacco group compared with the <1% spent group were 0.43 (0.18–1.03) for exclusive combustible cigarette smokers, 0.71 (0.20–2.54) for exclusive HTPs users, and 0.11 (0.02–0.77) for dual users.

**Conclusions:**

Higher tobacco expenditure was not associated with quit intention for all categories of tobacco product users, probably due to the low price of tobacco in Japan.

**Supplementary information:**

The online version contains supplementary material available at https://doi.org/10.1265/ehpm.22-00103.

## Graphical Abstract

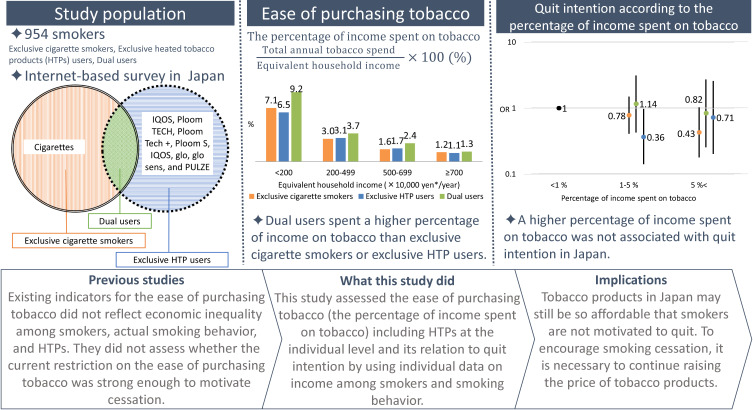

## 1. Introduction

It is a widely accepted fact that tobacco smoking has a negative impact on health and relates to economic costs. The World Health Organization (WHO) estimates that smoking is one of the world’s largest preventable causes of premature mortality, accounting 8 million deaths and costing the global economy US$ 1.4 trillion annually [1].

The ease of purchasing tobacco, expressed as expenditure on tobacco relative to income, is a key determinant of smoking behavior [2–6]. A high percentage of income spent on tobacco means that tobacco is difficult to purchase, and this encourages smoking cessation. To assess whether the ease of purchasing tobacco has been sufficiently reduced to encourage smoking cessation, it would be useful to evaluate the association between percentage of income spent on tobacco and quit intention.

Some previous studies on this topic have used Relative Income Price (RIP) as the percentage of income spent on tobacco, and used affordability elasticity as an indicator of how smoking consumption changed following a change in RIP. RIP was the percentage of per capita gross domestic product (GDP) needed to purchase 100 packs of cigarettes [7–9]. The higher the value of the RIP, the more difficult it is to purchase tobacco. The affordability elasticity was the change in smoking consumption in response to a change in RIP; for example, affordability elasticity of −0.4% indicates that if RIP increases by 10%, consumption will decrease by 4%.

A previous ecological study of 70 countries suggested that a 10% increase in RIP was expected to decrease tobacco consumption by between 4.9% and 5.7% [7]. A recent study of 78 countries suggested that a 10% increase in RIP would decrease tobacco consumption by 2.0% [8]. Another longitudinal study from China showed that a 10% increase in RIP was associated with a 1.7% decrease in tobacco consumption [9].

Previous studies have three limitations. First, RIP in most studies [7, 8] could not assess actual expenditure on tobacco because the researchers relied on average national estimates of income (GDP) and average tobacco price derived from the most popular brands (i.e., Marlboro). These ‘national-level’ indicators did not fully consider the differences in economic status among smokers, prices among tobacco products, and the actual amount smoked. Although one study from China estimated RIP by using smokers’ income and tobacco price paid, the actual amount smoked was not considered [9]. To overcome this limitation, “individual-level” indicators are needed which use individual data for smokers on income and on actual smoking activity (price paid for tobacco products, amount smoked, and expenditure on smoking).

Second, as discussed in one of the above studies as a limitation [9], the affordability elasticity merely predicts the change in smoking consumption in response to changes in RIP among those who keep smoking. It did not assess whether the current restriction on the ease of purchasing tobacco was strong enough to promote quitting because it was calculated as the regression coefficient of RIP as the predictor variable with per capita tobacco consumption as the response variable [7–9]. It is thus necessary to examine quit intention among smokers according to the percentage of income spent on tobacco.

Third, previous studies did not explain the difference in ease of purchase between different tobacco products such as combustible cigarettes, or heated tobacco products (HTPs). Previously, no indicators of ease of purchasing tobacco have included HTPs such as IQOS, Ploom TECH, and glo, which first became popular in Japan. Since the launch of IQOS in 2014, Japan has been the only country to have a national roll-out of IQOS and the country’s share of worldwide IQOS sales was 96% in October 2016 [10]. The components of mainstream smoke of HTPs are different from those of conventional cigarettes [11]. There are some differences in the profile between HTPs users and combustible cigarette users. The HTPs use tended to be more prominent among those with the higher economic status although cigarette use was likely to be higher in the lower economic status population (shown in Supplementary Table 1) [12].

In Japan it has been easy to purchase tobacco [7]. Although the tobacco price has increased over time and the smoking prevalence has declined in Japan [6], there is no consensus that the current situation regarding ease of purchasing tobacco is satisfactory for tobacco control in the population. Therefore, it is important to assess current ease of purchasing tobacco and its relation to quit intention.

In this study, we aimed to examine the percentage of income spent on tobacco at an individual level and its relation to quit intention among combustible cigarette and/or HTP users.

## 2. Methods

### 2.1. Data

We conducted a cross-sectional study using internet-based self-reported questionnaires from February–March 2020, as part of the Japan Society and New Tobacco Internet Survey (JASTIS) [13]. Participants were drawn from the registers of Rakuten Insight, one of Japan’s largest internet research companies which maintains a pool of 2.3 million panelists covering all social categories (eg, education level, housing tenure and marital status) defined by the census in Japan [14]. The survey panel consisted of people recruited initially through services managed by the Rakuten group. At registration, they were required to provide information such as sex, age, occupation, and place of residence and to agree to participate in different research surveys.

### 2.2. Participants

In 2020, 9,437 respondents participated in the study. Among them, we targeted 1,586 (representing 16.8% of the survey respondents) respondents who currently smoked. Since the latest current smoking prevalence in Japan in 2019 was 16.7% [15], this smoking prevalence among respondents in the present study was considered reasonable. Currently smoked respondents who met the following criteria were excluded: respondents who always chose the same number in an entire set of questions; (n = 146); those whose income was ‘0 yen’ or ‘missing’ (n = 256), those whose income was an outlier (n = 14); those whose ease of purchasing tobacco was calculated to be more than 100% (n = 2), and current smokers who answered the question about quit intention as: 1. ‘I have never smoked a cigarette (HTP)’ or 2. ‘I’m quitting smoking cigarettes (HTPs) now, and I have quit smoking cigarettes (HTPs) for more than 6 months’ (n = 214). After these exclusions, 954 smokers (representing 60% of the survey respondents who currently smoked) remained for analysis. Since the income data was skewed, we logarithmized the income and used Tukey’s definition of “far out” [first quartile − (3 × interquartile range) and third quartile + (3 × interquartile range)] as the outlier [16].

### 2.3. Measures

#### 2.3.1. Ease of purchasing tobacco

Ease of purchasing tobacco was calculated as the percentage of the annual equivalent household income that was spent on tobacco (equation 1) [17]; where higher values indicated difficulty in purchasing tobacco. This indicator also shows the magnitude of the financial burden of smoking; where higher values indicated greater financial burden.

$$\begin{array}{rl} & \text{Ease of purchasing tobacco}\\ & \;=\frac{\text{Total annual tobacco spend}}{\text{Equivalent household income}}\times 100\;(\%) \end{array}$$ (1)

#### 2.3.2. Current use (use in the previous 30 days)

Participants were asked about their current use (use in the previous 30 days) of each product (*cigarettes, IQOS, Ploom TECH, Ploom Tech +, Ploom S, IQOS, glo, glo sens, and PULZE*). Respondents who answered ‘yes’ to the question, “Have you used any of the following products in the previous 30 days?” (options: *cigarettes, IQOS, Ploom TECH, Ploom Tech +, Ploom S, IQOS, glo, glo sens, and PULZE*) were defined as current users of the designated product. As shown in Supplementary Figure 1, current users of cigarettes were defined as cigarette smokers. Current users of any of the listed HTP products were defined as HTP users. Dual users were defined as current cigarette smokers who concurrently used any HTP in the previous 30 days. Exclusive cigarette smokers were defined as current cigarettes users who had not concurrently used any HTP in the previous 30 days. Exclusive HTP users were defined as current HTP users who had not concurrently used cigarettes in the previous 30 days. Current users were asked ‘number smoked per day’ and ‘number of days smoked per month’ for each tobacco product.

#### 2.3.3. Annual tobacco spend

Current users were also asked: ‘How much is a pack of the tobacco products you most often use?’. Most often-used product was defined as the product the participant smoked most per day. Annual tobacco spend was calculated for each tobacco product as equation 2 and summed to give total annual tobacco expenditure.

$$\begin{array}{rl} & \text{Annual tobacco spend}\\ & \;=\frac{\text{Price per pack of tobacco product}}{\text{Number of products per pack}}\\ & \;\times \text{Number smoked per day}\\ & \;\times \text{Number of days of smoking per month}\times 12 \end{array}$$ (2)

The number of products per pack was defined as twenty.

If a tobacco product was regarded as the most-used product based on the number smoked per day, the price per pack of the tobacco product was taken from the response to the questionnaire. If the tobacco product was not regarded as the most-used product, the price used for a pack of the tobacco product was 490 yen for cigarettes and 500 yen for HTPs.

#### 2.3.4. Equivalent household income

Panelists were asked about their income as follows: ‘How much was your household annual income (including tax) last year? Please select the number that applies to you.’ Supplementary Table 2 shows the response options and converted responses. Equivalent household income per year was calculated by combining the income reported by the respondent and his/her spouse and dividing the sum of the income by the square root of the number of family members, in order to adjust for household size, and was categorized into 4 groups (range: lowest ≤2,000,000 yen; 2nd 2,000,000–4,990,000 yen; 3rd 5,000,000–6,990,000 yen; highest ≥7,000,000 yen). Each cut-off for the category was defined based on the approximate value of the poverty line (1,270,000 yen) [18], average family income (5,523,000 yen) [18], and the deemed high-income line (8,500,000 yen) [19] in Japan while taking the distribution of population in those classes into account.

#### 2.3.5. Quit intention

Quit intention was assessed using the following questions for cigarettes or HTPs, respectively [20]: ‘How interested are you in quitting smoking cigarettes (HTPs)?’. Seven response options and their classification were shown in Supplementary Table 3. For exclusive cigarette smokers, quit intention was defined as intention to quit smoking combustible cigarettes. For exclusive HTP smokers, quit intention was defined as intention to quit using HTPs. For dual users, four types of quit intention were defined; intention to quit cigarettes or HTPs, intention to quit both cigarettes and HTPs, intention to quit cigarettes, and intention to quit HTPs.

#### 2.3.6. Covariates

We converted variables of socio-demographic characteristics to categorical variables: sex (men, women), age (15–19, 20–29, 30–39, 40–49, 50–59, 60–72 years), time-to-first-use of tobacco in the morning (within 5 minutes, 6–15 minutes, 16–30 minutes, 31–60 minutes, and more than an hour), education (high school or below, college, university or above), marital status (married, never married, widowed/divorced), and self-rated health status (poor or not poor).

### 2.4. Statistical analysis

The percentage of income spent on tobacco and socio-demographic characteristics were calculated according to the equivalent household income category for each tobacco product user. The odds ratios (ORs) and 95% confidential intervals (95% CIs) for quit intention, according to three categories of percentage of income spent on tobacco (<1%, 1–5%, >5%), were calculated using logistic regression. We included sex, age, education, marital status, and self-rated health status as covariates.

Probability values for statistical tests were two tailed; p < 0.05 was considered statistically significant. The data analysis for this paper was done using SAS software, Version 9.4 of the SAS System for Microsoft Windows (SAS Institute Inc., Cary, NC, USA).

## 3. Results

Of the 954 analyzed total smokers, 538 were exclusive cigarettes smokers, 157 were exclusive HTP users, and 259 were dual users.

Table 1 shows the smoking and socio-demographic characteristics of each category of tobacco product user according to equivalent household income group. For every income group, annual tobacco spend and tobacco consumption per day were the largest among dual users. Average daily consumption of cigarettes in the lowest income groups was nine cigarettes for dual users, and 13 cigarettes for exclusive cigarette smokers; daily consumption of HTPs was seven products for dual users, and 11 products for exclusive HTPs users, while the total daily consumption of tobacco products was 16 (nine cigarettes and seven products) for dual users. The lower the income, the smaller the tobacco spend and the lower the price per pack, while more people smoked within 30 minutes of waking up among exclusive HTP users and dual users. Tobacco consumption did not differ by income group. Among exclusive HTP users and dual users, the lower the income group was, the younger the age was and the lower the educational status was. Among exclusive cigarette smokers and HTP users, the lower the income group was, the more women were and the poorer self-rated health was.

**Table 1:** Characteristics of tobacco product smokers according to equivalent household income

		**Equivalent household income (×10,000 yen/year)**			
	
	**Total**	**<200**	**200–499**	**500–699**	**≥700**
Exclusive combustible cigarette smoker, n (%)*	538 (100)	96 (17.8)	307 (57.1)	84 (15.6)	51 (9.5)
**Smoking characteristics**					
Annual tobacco spend (yen/year), mean (±SD)	95728 (71211)	92534 (65834)	95867 (72101)	91821 (65354)	107338 (84501)
Tobacco consumption (number smoked/day), mean (±SD)	13 (8)	13 (8)	13 (9)	12 (8)	14 (10)
Price per pack (yen), mean (±SD)	453 (64)	448 (72)	455 (60)	456 (60)	447 (72)
Time-to-first-smoke under 30 minutes, n (%)^†^	354 (65.8)	62 (64.6)	202 (65.8)	55 (65.5)	35 (68.6)
**Socio-demographic characteristics**					
Equivalent household income (×10,000 yen/year), mean (±SD)	407 (273)	141 (35)	340 (84)	582 (51)	1020 (389)
Age (years), mean (±SD)	49.4 (14.2)	50.6 (15.2)	49.1 (14.5)	50.0 (12.7)	47.4 (12.0)
Sex (Female), n (%)^†^	138 (25.7)	26 (27.1)	83 (27.0)	17 (20.2)	12 (23.5)
College or higher education, n (%)^†^	249 (46.3)	29 (30.2)	146 (47.6)	44 (52.4)	30 (58.8)
Poor self-rated health, n (%)^†^	64 (11.9)	21 (21.9)	32 (10.4)	8 (9.5)	3 (5.9)

Exclusive heated tobacco product user, n (%)*	157 (100)	19 (12.1)	90 (57.3)	25 (15.9)	23 (14.7)
**Smoking characteristics**					
Annual tobacco spend (yen/year), mean (±SD)	99166 (78204)	85468 (102162)	102291 (75442)	99135 (80963)	98288 (66704)
Tobacco consumption (number smoked/day), mean (±SD)	13 (9)	11 (11)	13 (9)	12 (9)	15 (10)
Price per pack (yen), mean (±SD)	480 (41)	444 (65)	482 (35)	496 (31)	482 (30)
Time-to-first-smoke under 30 minutes, n (%)^†^	103 (65.6)	14 (73.7)	58 (64.4)	16 (64.0)	15 (65.2)
**Socio-demographic characteristics**					
Equivalent household income (×10,000 yen/year), mean (±SD)	450 (298)	148 (34)	343 (85)	579 (46)	975 (405)
Age (years), mean (±SD)	43.4 (11.8)	38.9 (12.2)	43.3 (11.1)	42.7 (12.9)	48.7 (11.6)
Sex (Female), n (%)^†^	41 (26.1)	11 (57.9)	21 (23.3)	4 (16.0)	5 (21.7)
College or higher education, n (%)^†^	78 (49.7)	4 (21.1)	42 (46.7)	15 (60.0)	17 (73.9)
Poor self-rated health, n (%)^†^	26 (16.6)	4 (21.1)	18 (20.0)	2 (8.0)	2 (8.7)

Dual user of combustible cigarettes and heated tobacco products, n (%)*	259 (100)	39 (15.1)	140 (54.1)	45 (17.4)	35 (13.5)
**Smoking characteristics**					
Annual tobacco spend (yen/year), mean (±SD)	120416 (77523)	103227 (86981)	121498 (74325)	136730 (90055)	114269 (57734)
Tobacco consumption (number of combustible cigarettes smoked/day), mean (±SD)	10 (8)	9 (8)	10 (8)	10 (10)	11 (6)
Tobacco consumption (number of heated tobacco product smoked/day), mean (±SD)	7 (8)	7 (11)	7 (7)	8 (7)	5 (5)
Price per pack (yen), mean (±SD)	463 (51)	446 (56)	466 (50)	477 (34)	455 (59)
Time-to-first-smoke under 30 minutes, n (%)^†^	215 (68.9)	33 (84.6)	93 (66.4)	32 (71.1)	22 (62.9)
**Socio-demographic characteristics**					
Equivalent household income (×10,000 yen/year), mean (±SD)	426 (244)	118 (45)	342 (81)	586 (53)	897 (134)
Age (years), mean (±SD)	42.8 (14.8)	42.2 (18.3)	41.5 (14.0)	44.2 (15.2)	47.0 (12.6)
Sex (Female), n (%)^†^	55 (21.2)	9 (23.1)	32 (22.9)	6 (13.3)	8 (22.9)
College or higher education, n (%)^†^	142 (54.8)	19 (48.7)	71 (50.7)	29 (64.4)	23 (65.7)
Poor self-rated health, n (%)^†^	24 (9.3)	5 (12.8)	16 (11.4)	3 (6.7)	0 (0.0)

*(%) indicates the percentage of people who belong to each equivalent household income group when the number of people in the total column is assigned as 100%.

^†^(%) indicates the percentage of people with the corresponding characteristics among those in each equivalent household income group.

Table 2 shows the percentage of income spent on tobacco according to income group. The lower the income group, the higher the percentage of income spent on tobacco; the percentage of income spent on tobacco in the lowest/highest income group was 7.1%/1.2% for exclusive cigarette smokers; 6.5%/1.1% for exclusive HTP users; and 9.2%/1.3% for dual users. In every income group, dual users spent the largest share of their income on tobacco. The percentage of income spent on tobacco was almost the same among exclusive cigarette smokers and exclusive HTP users. As shown in Supplementary Tables 4 and 5, these results were almost consistent by sex and age, with some exceptions; male exclusive HTP users spent the largest share of their income on tobacco in the lowest income group (9.5%), and exclusive HTP users spent more of their income on tobacco than exclusive cigarette smokers at age 49 and younger, and vice versa at age 50 and older.

**Table 2:** Percentage of income spent on tobacco according to equivalent household income

		Equivalent household income (×10,000 yen*/year)			
	
	**Total**	**<200**	**200–499**	**500–699**	**≥700**
**Exclusive combustible cigarette smokers, n**	538	96	307	84	51
Percentage of income spent on tobacco (%), mean (±SD)	3.4 (3.7)	7.1 (6.0)	3.0 (2.5)	1.6 (1.2)	1.2 (1.0)
**Exclusive heated tobacco product users, n**	157	19	90	25	23
Percentage of income spent on tobacco (%), mean (±SD)	3.0 (3.8)	6.5 (8.1)	3.1 (2.7)	1.7 (1.5)	1.1 (0.8)
**Dual users of combustible cigarette and heated tobacco product, n**	259	39	140	45	35
Percentage of income spent on tobacco (%), mean (±SD)	4.0 (4.5)	9.2 (8.4)	3.7 (2.5)	2.4 (1.7)	1.3 (0.7)

*10,000 yen = 91.8 US dollar

Table 3 shows quit intention according to the percentage of income spent on tobacco. Smokers who spent >5% of their income on tobacco did not have more quit intention than smokers who spent <1% of their income; multivariable adjusted ORs and 95% CIs of quit intention for smokers with >5% spent on tobacco was 0.43 (0.18–1.03) for exclusive cigarette smokers and 0.71 (0.20–2.54) for exclusive HTP users. Among dual users with >5% spent on tobacco, quit intention for cigarettes or HTPs was 0.82 (0.25–2.68), 0.11 (0.02–0.77) for dual use, 0.35 (0.09–1.37) for cigarettes, and 0.63 (0.16–2.51) for HTPs, respectively.

**Table 3:** Intention to quit smoking according to percentage of income spent on tobacco

	**Percentage of income spent on tobacco**		

	**<1%**	**1–5%**	**5%<**
**Exclusive combustible cigarette smokers**			
No of participants	141	279	118
Intention to quit smoking combustible cigarettes, n	25	41	11
Multivariable OR (95%CI)*	1.0 (reference)	0.78 (0.41–1.50)	0.43 (0.18–1.03)

**Exclusive heated tobacco product users**			
No of participants	45	88	24
Intention to quit smoking HTPs, n	15	13	6
Multivariable OR (95%CI)*	1.0 (reference)	0.36 (0.14–0.96)	0.71 (0.20–2.54)

**Dual users on combustible cigarette and heated tobacco product**			
No of participants	34	166	59
Intention to quit smoking combustible cigarettes or HTPs, n	7	33	11
Multivariable OR (95%CI)*	1.0 (reference)	1.14 (0.42–3.07)	0.82 (0.25–2.68)
Intention to quit smoking combustible cigarette and HTPs, n	5	14	2
Multivariable OR (95%CI)*	1.0 (reference)	0.43 (0.12–1.60)	0.11 (0.02–0.77)
Intention to quit smoking combustible cigarettes, n	7	26	6
Multivariable OR (95%CI)*	1.0 (reference)	0.80 (0.28–2.29)	0.35 (0.09–1.37)
Intention to quit smoking HTPs, n	5	21	7
Multivariable OR (95%CI)*	1.0 (reference)	0.90 (0.29–2.82)	0.63 (0.16–2.51)

*Multivariable adjusted for age, sex, time-to-first-use, educational status, and self-rated health.

## 4. Discussion

The present study revealed that (1) the percentage of income spent on tobacco was approximately 5–7 times higher among low-income smokers than among high-income smokers, (2) dual users spent a higher percentage of their income on tobacco than exclusive cigarette smokers or exclusive HTP users, and (3) a higher percentage of income spent on tobacco was not associated with quit intention, contrary to the theory that the financial burden of smoking encourages cessation. This is the first study to evaluate the ease of purchasing tobacco for HTPs.

The finding that low-income smokers spend a higher percentage of income on tobacco than high-income smokers was consistent with the results from previous similar studies [17, 21–23]. In the present study, this disparity is due to the large difference in household income levels because both annual tobacco spend and price per pack of tobacco products were lower in lower-income groups than in higher-income groups.

The higher percentage of income spent on tobacco among dual users would be attributable to a larger amount of total tobacco consumption. Although the respective consumption of cigarettes and HTPs among dual users was smaller than that among exclusive cigarettes smokers or exclusive HTPs users, their total consumption was higher.

Even though there is no consensus whether HTPs help smoking cessation [24], they have been marketed as an aid for cessation. In fact, HTP users who switched from cigarettes to HTPs have been reported to increase their HTP use in terms of both frequency and amount [25]. Also, cigarette smokers do not necessarily switch to HTPs. According to an Internet survey conducted in Japan, 72% of HTP users also concurrently used cigarettes [26].

A cross-sectional study with 859 smokers in the UK showed similar results about tobacco expenditure among dual users. Dual users of cigarettes and alternative nicotine products including e-cigarettes spent more on smoking products (£24.54 per week for cigarettes and £7.49 for alternative nicotine products) than exclusive cigarettes smokers (£23.09) and exclusive alternative nicotine product users (£8.59) [27]. In Japan, e-cigarettes are regarded as one of the new types of cigarettes as well as HTPs [10].

Previous studies of lower-middle income countries reported that individuals with lower income and dual users prominently reduced the expenditure on other fundamental commodities such as food, health, education etc. due to the higher tobacco spending, known as the crowding-out effect [28, 29]. Our results on the higher percentage of income spent on tobacco among lower-income smokers and dual users might suggest that tobacco spending could be a burden on expenditure on basic goods among such smokers even in high-income countries like Japan.

The present study showed that higher percentage of income spent on tobacco was not associated with quit intention. This result was inconsistent with previous results showing that a 10% greater difficulty in purchasing tobacco reduces smoking consumption by about 1.7–5.7% [7–9].

The “national-level” indicator of the ease of purchasing tobacco has been standardized for comparison between countries and for assessing long-term trends within one country. Therefore, it does not fully reflect the actual tobacco consumption of smokers or their economic status. Also, previous studies examined how the ease of purchasing tobacco affected smoking cessation behavior by assessing changes in tobacco consumption, not whether smokers quit smoking. The present study expanded on previous studies by using an “individual-level” indicator of the ease of purchasing tobacco and quit intention. This indicator was calculated using smokers’ actual smoking behavior (price of tobacco, number of cigarettes/HTPs smoked) and income. The present study showed that higher percentage of income spent on tobacco was not associated with quit intension, that is, the current restriction on the ease of purchasing tobacco has not produced an adequate level of smoking cessation.

Due to the cross-sectional design of this study, we could not determine the causal direction between the percentage of income spent on tobacco and quit intention. However, the following possibilities can be suggested.

First, tobacco products in Japan may still be so affordable that smokers are not motivated to quit. In Japan, a special tobacco tax was imposed in 1998 and the real price of tobacco increased in 2003, 2006, 2010, 2014, 2016, 2018, and 2019. The price of a pack of the most popular brand in Japan, Mild Seven (the brand name was changed to ‘Mevius’ in 2013), increased from 250 to 270 yen in 2003, to 300 yen in 2006, to 410 yen in 2010, to 430 yen in 2014, to 440 yen in 2016, to 480 yen in 2018, and to 490 yen in 2019 [30]. However, the price of tobacco in Japan remains one of the lowest in the world; the percentage of GDP per capita required to purchase 2000 cigarettes of the highest-selling brand was 0.74% in 2008, 0.77% in 2010, 1.06% in 2012, 1.06% in 2014, 1.04% in 2016, and 1.00% in 2018 [31]. The corresponding percentages in 2018 were 2.11% for high-income countries, 6.24% for middle-income countries and 17.9% for low-income countries (calculated by the authors in accordance with reference 31).

Second, smokers without quit intention may be able to keep spending their income on tobacco products. It is well known that smokers change tobacco purchasing behaviors in response to price increases; for example, switching to cheaper brands or buying from low or untaxed sources [32, 33]. These price minimizing strategies tend to be adopted by smokers with lower socio-economic status [34]. Large price differentials between discount and premium brands promote the switching strategy. In Japan, the price gap between most expensive and cheapest brands was 70.2% [31]. Because current smokers in the present study were probably persistent smokers who continued to smoke despite past price rises, they might use price minimizing strategies and maintain their smoking expenditure. An implication from the present study is that current tobacco pricing in Japan is too low to adequately control tobacco consumption.

The present study has several limitations. First, the results were obtained through a self-reported internet questionnaire survey. Although the polling company aims to achieve representativeness, the distribution of the population might be imperfect. As the information was self-reported, we excluded respondents with discrepancies or inconsistencies in their answers. However, since the true effects and implications of HTP use are unknown, we could not confirm the validity of the exclusion. Second, the data were collected from a mainly Japanese population. Because tobacco price setting and income levels differ from country to country, careful interpretation of our findings is needed. Nevertheless, we believe that the trend of a higher percentage of income spent on tobacco among dual users observed in the present study is generalizable. Third, the data were not weighted to population, and therefore the results might differ from the actual proportions. Fourth, due to the cross-sectional design of the study, we cannot surmise whether a higher percentage of income spent on tobacco led to lower quit intention, or lower quit intention led to a higher percentage of income spent on tobacco, or both. It is necessary a future investigation by a prospective cohort study on this issue. Fifth, there was no information about the price of the devices which were necessary to use HTPs. The lack of this information might underestimate the percentage of income spent on tobacco among exclusive HTPs users and dual users. Future examination considering the price of the devices would contribute to the more precise estimation for the percentage of income spent on tobacco. Finally, the percentage of very high income spent of tobacco, equivalent to low-income countries (e.g., more than 15%) [7] in this population was only 1.5% for exclusive cigarettes smokers, 1.9% for exclusive HTPs users, and 3.0% for dual users. This may have caused the percentage of income spent on tobacco not to be associated with quit intention.

## 5. Conclusion

The percentage of income spent on tobacco was higher among dual users, who account for the majority of HTP users, than exclusive combustible cigarette or HTP users.

To encourage smoking cessation, it is necessary to continue raising the price of tobacco products in Japan where these products are still easy to purchase.

## Abbreviations

HTP: heated tobacco product
WHO: World Health Organization
RIP: Relative Income Price
GDP: gross domestic product
JASTIS: the Japan Society and New Tobacco Internet Survey
OR: odds ratio
95% CI: 95% confidential interval

## Supplementary information

**Additional file 1:** **Supplementary Figure 1.** The definition of current tobacco products use.

**Additional file 2:** **Supplementary Table 1.** Proportions of various types of smokers by annual household income. **Supplementary Table 2.** Response options for household annual income in the questionnaire and converted response. **Supplementary Table 3.** Response options for quit intention in the questionnaire and categoroized response. **Supplementary Table 4.** Percentage of income spent on tobacco according to equivalent household income by sex. **Supplementary Table 5.** Percentage of income spent on tobacco according to equivalent household income by age.

## Declarations

### Availability of data and materials

The data used in this study are not available in a public repository because they contain personally identifiable or potentially sensitive participants’ information. Based on the regulations for ethical guidelines in Japan, the Research Ethics Committee of the Osaka International Cancer Institute has imposed restrictions on the dissemination of the data collected in this study. All data inquiries will be channeled through Tabuchi (moc.liamg@katihcubat) to Osaka Cancer Institute Institutional Ethics Committee.
