# Supplementary material for: Percentage of income spent on tobacco and intention to quit: a cross-sectional analysis of the JASTIS 2020 study
Source: Environ Health Prev Med. 2022 Dec 3;27:46. doi: 10.1265/ehpm.22-00103 (PMC9761199; doi:10.1265/ehpm.22-00103)
Supplement: Supplementary file 1 — Additional file 1: Supplementary Figure 1. The definition of current tobacco products use. [file ehpm-27-046-s001.pptx]

## Slide 1
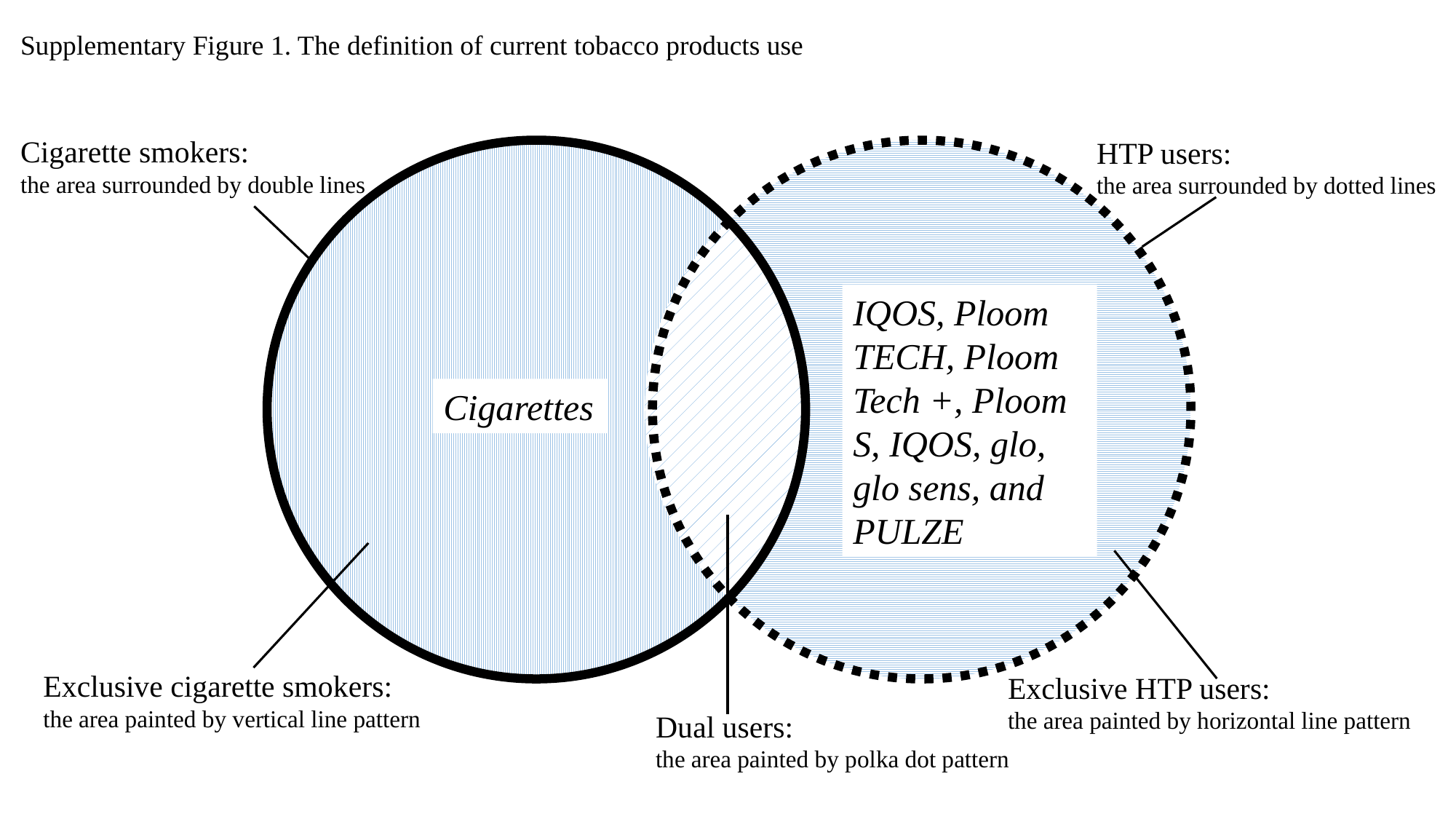

Supplementary Figure 1. The definition of current tobacco products use
Cigarette smokers:
the area surrounded by double lines
HTP users:
the area surrounded by dotted lines
IQOS, Ploom TECH, Ploom Tech +, Ploom S, IQOS, glo, glo sens, and PULZE
Cigarettes
Exclusive cigarette smokers:
the area painted by vertical line pattern
Exclusive HTP users:
the area painted by horizontal line pattern
Dual users:
the area painted by polka dot pattern
