# Supplementary material for: Percentage of income spent on tobacco and intention to quit: a cross-sectional analysis of the JASTIS 2020 study
Source: Environ Health Prev Med. 2022 Dec 3;27:46. doi: 10.1265/ehpm.22-00103 (PMC9761199; doi:10.1265/ehpm.22-00103)
Supplement: Supplementary file 2 [file ehpm-27-046-s002.docx]

Supplementary Table 1. Proportions of various types of smokers by annual household income

	Annual household income (× 10,000 yen*/year)			
	<400	400-800	≥800	No response
**Men**				
Any tobacco use past 30 days, % (95 %CI)	29.8 (27.9-31.7)	36.3 (34.3-38.3)	25.5 (23.6-27.4)	23.8 (22.0-25.6)
HTPs user, lifetime, % (95 %CI)	9.0 (7.8-10.2)	17.0 (15.4-18.6)	14.4 (12.9-15.9)	9.2 (8.0-10.4)
HTPs user, past 30 days, % (95 %CI)	4.4 (3.5-5.3)	11.3 (10.0-12.6)	8.4 (7.2-9.6)	4.9 (4.0-5.8)
**Women**				
Any tobacco use past 30 days, % (95 %CI)	12.5 (11.2-13.8)	9.8 (8.6-11.0)	7.5 (6.5-8.5)	7.5 (6.5-8.5)
HTPs user, lifetime, % (95 %CI)	3.5 (2.8-4.2)	4.7 (3.9-5.5)	3.1 (2.4-3.8)	3.0 (2.3-2.7)
HTPs user, past 30 days, % (95 %CI)	1.6 (1.1-2.1)	2.5 (1.9-3.1)	1.5 (1.0-2.0)	1.6 (1.1-2.1)

*Edited by the authors based on reference 12.

Supplementary Table 2. Response options for household annual income in the questionnaire and converted response

Q. How much was your household annual income (including tax) last year? Please select the number that applies to you.			
Response options in the questionnaire			Converted response
1	no income	→	0 yen
2	≤500,000 yen	→	250,000 yen
3	500,000-1,000,000 yen	→	750,000 yen
4	1,000,000-2,000,000 yen	→	1,500,000 yen
5	2,000,000-3,000,000 yen	→	2,500,000 yen
6	3,000,000-4,000,000 yen	→	3,500,000 yen
7	4,000,000-5,000,000 yen	→	4,500,000 yen
8	5,000,000-6,000,000 yen	→	5,500,000 yen
9	6,000,000-7,000,000 yen	→	6,500,000 yen
10	7,000,000-8,000,000 yen	→	7,500,000 yen
11	8,000,000-9,000,000 yen	→	8,500,000 yen
12	9,000,000-10,000,000 yen	→	9,500,000 yen
13	10,000,000-12,000,000 yen	→	11,000,000 yen
14	12,000,000-14,000,000 yen	→	13,000,000 yen
15	14,000,000-16,000,000 yen	→	15,000,000 yen
16	16,000,000-18,000,000 yen	→	17,000,000 yen
17	18,000,000-20,000,000 yen	→	19,000,000 yen
18	≥20,000,000 yen	→	25,000,000 yen
19	I do not want to answer.	→	missing
20	I do not know.	→	missing

Supplementary Table 3. Response options for quit intention in the questionnaire and categoroized response

Q. How interested are you in quitting smoking cigarettes (HTPs)?			
Response options in the questionnaire			Classification of response
1	I’ve never smoked a cigarette (HTPs).	→	Exclusion criteria met
2	I'm quitting smoking cigarettes (HTPs) now, and I've quit smoking cigarettes (HTPs) for more than 6 months.	→	Exclusion criteria met
3	I've quit smoking cigarettes (HTPs) now, but for less than 6 months.	→	Exclusion criteria met
4	I’m not interested in quitting smoking cigarettes (HTPs).	→	No quit intention for smoking cigarettes (HTPs)
5	I'm interested in quitting smoking cigarettes (HTPs), but I'm not thinking of quitting in the next 6 months.	→	No quit intention for smoking cigarettes (HTPs)
6	I'm thinking of quitting smoking cigarettes (HTPs) within the next 6 months, but I have no intention of quitting within the next month.	→	Having quit intention for smoking cigarettes (HTPs)
7	I'm thinking of quitting smoking cigarettes (HTPs) within the next month.	→	Having quit intention for smoking cigarettes (HTPs)

Supplementary Table 4. Percentage of income spent on tobacco according to equivalent household income by sex

		Equivalent household income (× 10,000 yen*/year)			
	Total	<200	200-499	500-699	≥700
**Men**					
**Exclusive combustible cigarette smokers, n**	400	70	224	67	39
Percentage of income spent on tobacco (%), mean (±SD)	3.4 (3.7)	7.1 (5.7)	3.1 (2.7)	1.8 (1.2)	1.3 (1.1)
**Exclusive heated tobacco product users, n**	116	8	69	21	18
Percentage of income spent on tobacco (%), mean (±SD)	3.1 (4.1)	9.5 (11.1)	3.3 (2.8)	1.7 (1.5)	1.2 (0.7)
**Dual users of combustible cigarette and heated tobacco product, n**	204	30	108	39	27
Percentage of income spent on tobacco (%), mean (±SD)	4.0 (4.2)	8.7 (7.7)	4.0 (2.6)	2.4 (1.7)	1.4 (0.8)
**Women**					
**Exclusive combustible cigarette smokers, n**	138	26	83	17	12
Percentage of income spent on tobacco (%), mean (±SD)	3.2 (3.8)	7.3 (6.7)	2.7 (1.8)	1.0 (0.9)	0.8 (0.6)
**Exclusive heated tobacco product users, n**	41	11	21	4	5
Percentage of income spent on tobacco (%), mean (±SD)	2.8 (2.8)	4.3 (4.3)	2.7 (2.0)	1.7 (1.3)	0.9 (0.9)
**Dual users of combustible cigarette and heated tobacco product, n**	55	9	32	6	8
Percentage of income spent on tobacco (%), mean (±SD)	3.8 (5.5)	11.0 (10.9)	2.7 (1.8)	2.0 (1.3)	1.1 (0.5)

*10,000yen=91.8 US dollar

Supplementary Table 5. Percentage of income spent on tobacco according to equivalent household income by age

		Equivalent household income (× 10,000 yen*/year)			
	Total	<200	200-499	500-699	≧700
**Under 49 years old**					
**Exclusive combustible cigarette smokers, n**	252	39	151	35	27
Percentage of income spent on tobacco (%), mean (±SD)	2.7 (3.4)	5.9 (6.6)	2.5 (2.2)	1.4 (1.2)	1.0 (0.8)
**Exclusive heated tobacco product users, n**	108	16	64	20	8
Percentage of income spent on tobacco (%), mean (±SD)	3.3 (4.4)	7.1 (8.7)	3.2 (2.9)	1.7 (1.5)	0.8 (0.8)
**Dual users of combustible cigarette and heated tobacco product, n**	167	23	101	28	15
Percentage of income spent on tobacco (%), mean (±SD)	4.0 (4.8)	9.3 (10.1)	3.7 (2.6)	2.0 (1.2)	1.1 (0.8)
**Over 50 years old**					
**Exclusive combustible cigarette smokers, n**	286	57	156	49	24
Percentage of income spent on tobacco (%), mean (±SD)	3.9 (3.8)	8.0 (5.4)	3.5 (2.7)	1.8 (1.2)	1.3 (1.2)
**Exclusive heated tobacco product users, n**	49	3	26	5	15
Percentage of income spent on tobacco (%), mean (±SD)	2.4 (1.9)	3.1 (1.4)	3.1 (2.1)	1.7 (1.3)	1.3 (0.7)
**Dual users of combustible cigarette and heated tobacco product, n**	92	16	39	17	20
Percentage of income spent on tobacco (%), mean (±SD)	4.0 (3.8)	9.2 (5.5)	3.7 (2.3)	2.9 (2.2)	1.5 (0.7)

*10,000yen=91.8 US dollar
